# Anti-Inflammatory Effects of High Hydrostatic Pressure Extract of Mulberry (*Morus alba*) Fruit on LPS-Stimulated RAW264.7 Cells

**DOI:** 10.3390/molecules24071425

**Published:** 2019-04-11

**Authors:** Sunyoon Jung, Mak-Soon Lee, Ae-Jin Choi, Chong-Tai Kim, Yangha Kim

**Affiliations:** 1 Department of Nutritional Science and Food Management, Ewha Womans University, Seoul 03760, Korea; cococosy@naver.com (S.J.); troph@hanmail.net (M.-S.L.); 2 Functional Food & Nutrition Division, National Institute of Agricultural Science (NIAS), Rural Development Administration (RDA), Wanju 55365, Korea; aejini77@korea.kr; 3 R&D Center, EastHill Corporation, Gwonseon-gu, Suwon-si, Gyeonggi-do 16642, Korea; ctkim@ieasthill.com

**Keywords:** mulberry fruit, high hydrostatic pressure, macrophage, inflammation

## Abstract

Mulberry fruit (*Morus alba* L.) contains abundant bioactive compounds, including anthocyanins and flavonols, and has been reported to possess potent beneficial properties including anticancer, antidiabetic, and anti-oxidant effects. High hydrostatic pressure (HHP) processing, a nonthermal food processing technology, is suitable for the extraction of bioactive compounds from plants. Nevertheless, the anti-inflammatory effects of HHP extract of mulberry fruit (HM) in RAW264.7 cells remain unclear. The present study aimed to investigate the anti-inflammatory effects of HM on lipopolysaccharide (LPS)-induced inflammation *in vitro*. RAW264.7 cells were treated with various concentrations (0.1–1 μg/mL) of HM in the presence or absence of LPS. HM inhibited the inflammatory mediator, nitric oxide (NO) release, and mRNA expression of nitric oxide synthase 2 (NOS2) in LPS-induced RAW264.7 cells. In addition, HM suppressed both mRNA and protein expressions of prostaglandin-endoperoxide synthase 2 (PTGS2). Moreover, it reduced the LPS-induced secretion of proinflammatory cytokines such as interleukin (IL)-6 and tumor necrosis factor (TNF)-α. These results revealed that HM exerts anti-inflammatory effects by inhibiting several mediators and cytokines involved in the inflammatory process.

## 1. Introduction

Inflammation is a normal protective response to irritation, injury, and infection, and is required to maintain homeostasis of the immune system and for healing; however, it can damage the body if it is not regulated after a certain time period. Uncontrolled or prolonged inflammatory responses are often involved in the onset of chronic diseases, such as cancer, rheumatoid arthritis, and vascular diseases. Nonsteroidal anti-inflammatory drugs (NSAIDs) are commonly used in the treatment of inflammatory disease; however, currently available NSAIDs present serious side effects such as gastric lesions, bronchospasm, and kidney and cardiac damage [1]. Therefore, several studies have been conducted to find new anti-inflammatory agents without side effects as alternatives to NSAIDs.

Mulberry (*Morus alba* L.) is a flowering plant that belongs to the family Moraceae. Mulberry fruits are generally consumed due to their delicious taste, pleasing color, and high nutrient content [2]. Mulberry fruit contains various biologically active compounds, most of which are flavonoids, including anthocyanins and flavonols [3,4]. In Korea, China, and Japan, mulberry fruit is also used in folk remedies for its pharmacological effects, including fever reduction, sore throat treatment, liver and kidney protection, vision improvement, and blood pressure lowering ability [3]. Several studies have reported that mulberry fruit extracts possess a variety of biological activities, such as antioxidant [5], antidiabetic [6], antitumor [7], and immunomodulatory effects [8,9,10]. In addition, combined mulberry fruit and leaf extract (500 mg/kg body weight) improved delayed wound closure through regulation of NLR family pyrin domain containing 3 (NLRP3) inflammasome in high-fat diet-induced obese mice [8]. Phenolic extract of mulberry fruit phenolic extract (250 μg/mL) diminished Th1/Th2 cytokine secretion ratio in lipopolysaccharide (LPS)-stimulated mouse splenocytes [9]. Moreover, anthocyanins from *Solanum tuberosum* L. exerted anticancer activities through the modulation of protein Kinase B (Akt)-mTOR signaling [10]. Similarly, mulberry anthocyanins inhibited Akt-phosphoinositide 3-kinase (PI3K) pathways and reduced migration of B16-F1 melanoma cells [11]. Bilancio et al. have reported that inhibition of p110δ PI3K reduces inflammatory cell infiltration and alleviates arterial injury-induced restenosis, thus modulation of the PI3K pathway can be regarded as a therapeutic target in inflammation [12].

High hydrostatic pressure (HHP) is a nonthermal food processing technology, which uses a pressure of 100 MPa or more, and is used for plant material extraction and pasteurization [13]. Conventional thermal treatment for food extraction and sterilization damages the sensory and nutritional qualities of food via chemical reactions. Nevertheless, the HHP process can ensure safe, high quality foods while minimizing the damage to sensitive bioactive compounds [13]. Wang et al. have reported that HHP processing effectively preserves the total phenolic content and ensures the microbiological safety of mulberry juice as in the heat treatment process [14], which indicates that HHP extract of mulberry fruit may have the potential to be used as a health functional food.

Mulberry fruit dichloromethane extract presented a dose-dependent inhibition of lipopolysaccharide (LPS)-induced inflammatory reactions in macrophages [15]. Mulberry fruit polysaccharides isolated by ethanol exerted anti-inflammatory effects on LPS-stimulated macrophages by modulating the pro- and anti-inflammatory cytokines [16]; however, the effect of HHP extract of mulberry fruit (HM) on LPS-induced inflammation in vitro remains unclear. In the present study, we investigated the anti-inflammatory effect of HM in LPS-treated RAW264.7 macrophages. We evaluated the production of nitric oxide (NO) and the expression of nitric oxide synthase 2 (NOS2) and prostaglandin-endoperoxide synthase 2 (PTGS2), which are involved in the synthesis of inflammatory mediators. In addition, we analyzed the secretion of proinflammatory cytokines such as interleukin (IL)-6 and tumor necrosis factor (TNF)-α.

## 2. Results and Discussion

### 2.1. Content of Anthocyanins and Flavonols in HM

**Figure 1:**
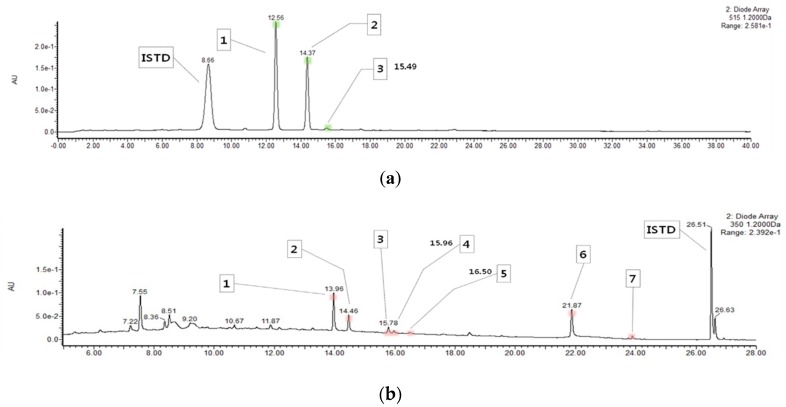
UPLC chromatograms of anthocyanins and flavonols in HM samples. (**a**) Anthocyanins (detected at 515 nm). ISTD, internal standard (cyanidin 3,5-diglucoside 100 ppm); Peak 1, cyanidin 3-*O*-glucoside (*m*/*z* 449); Peak 2, cyanidin 3-*O*-rutinoside (*m*/*z* 595); Peak 3, pelargonidin 3-*O*-glucoside (*m*/*z* 443). (**b**) Flavonols (detected at 350 nm). ISTD, internal standard (galangin 20 ppm); Peak 1, quercetin 3-*O*-rutinoside (rutin; *m*/*z* 610); Peak 2, quercetin 3-*O*-glucoside (isoquercitrin; *m*/*z* 464); Peak 3, quercetin 3-*O*-(6″-*O*-malonyl)glucoside (*m*/*z* 550); Peak 4, kaempferol 3-*O*-rutinoside (nicotiflorin; *m*/*z* 594); Peak 5, kaempferol 3-*O*-glucoside (astragalin; m/z 448); Peak 6, quercetin (*m*/*z* 302); Peak 7, kaempferol (*m*/*z* 286). UPLC, ultra-performance liquid chromatography; HM, high hydrostatic pressure extract of mulberry fruit.

Mulberry fruit is a rich source of anthocyanins and flavonoids, in particular flavonols. However, these flavonoids are sensitive to heat and are often lost during extraction; hence, it is necessary to apply an appropriate processing method to maintain its bioactivity while extracting flavonoids from mulberry fruit. HHP has been reported to be a suitable food processing technique for extracting bioactive compounds from plants as it enhances biological activity by improving mass transfer and preserving heat-sensitive bioactive compounds [13,17]. Therefore, we determined the contents of anthocyanins and flavonols of HM via ultra-performance liquid chromatography-photodiode array detector-quadrupole/time of flight-mass spectrometer (UPLC-PDA-Q/TOF-MS) analysis and investigated its anti-inflammatory effect in an in vitro model of inflammation. Interestingly, this is the first report on the polyphenolic profiling of HM. From the analysis, three anthocyanins, such as cyanidin 3-*O*-glucoside, cyanidin 3-*O*-rutinoside, and pelargonidin 3-*O*-glucoside, and seven flavonoids, including quercetin 3-*O*-rutinoside (rutin), quercetin 3-*O*-glucoside (isoquercitrin), quercetin 3-*O*-(6’’-O-malonyl)glucoside, kaempferol 3-*O*-rutinoside (nicotiflorin), kaempferol 3-*O*-glucoside (astragalin), quercetin, and kaempferol, were detected on the chromatograms of HM, as shown in Figure 1. The total anthocyanin content of HM was 127.15 ± 4.73 mg/100 g, as shown in Table 1. Among the anthocyanins isolated from HM, cyanidin 3-*O*-glucoside and cyanidin 3-*O*-rutinoside were found to be the most abundant (75.85 ± 2.79 and 49.08 ± 1.92 mg/100 g, respectively), as shown in Table 1. In contrast, the content of total flavonols of the HM was 26.79 ± 0.94 mg/100 g, as shown in Table 2. Of the seven flavonoids isolated from HM, quercetin and quercetin 3-*O*-rutinoside (12.99 ± 0.45 and 8.05 ± 0.19, respectively) were identified as the main flavonols in the HM, as shown in Table 2.

### 2.2. Effects of HM on Cell Viability

**Figure 2:**
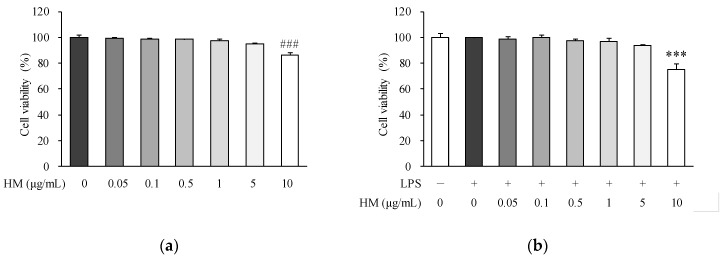
Effect of HM on cell viability in RAW264.7 cells. Cells were treated (**a**) without or (**b**) with 1 μg/mL of LPS and HM (0.05, 0.1, 0.5, 1, 5, and 10 μg/mL) for 24 h. Cell viability was assessed using the water-soluble tetrazolium salt (WST)-8 assay. Values are expressed as mean ± standard error of the mean (SEM) (*n* = 3). ^###^ *p* < 0.001 vs. untreated. *** *p* < 0.001 vs. LPS-treated control. LPS, lipopolysaccharide; HM, high hydrostatic pressure extract of mulberry fruit.

Prior to evaluating the effect of HM on LPS-induced inflammation in RAW264.7 cells, we performed a cytotoxic assay to select the appropriate concentration of HM. Cells were incubated with increasing concentrations of HM (0, 0.05, 0.1, 0.5, 1, 5, and 10 μg/mL) with or without LPS for 24 h. At concentrations below 5 μg/mL, HM did not change the cell viability compared to the untreated control, as shown in Figure 2a. Moreover, similar results were observed in the LPS-stimulated cells. When the cells were incubated with LPS, HM below 5 g/mL did not affect the cell viability compared to the LPS control, as shown in Figure 2b. Therefore, the concentration of 0.1–1 μg/mL was selected for further experiments.

### 2.3. Effects of HM on NO Production and NOS2 mRNA Expression

**Figure 3:**
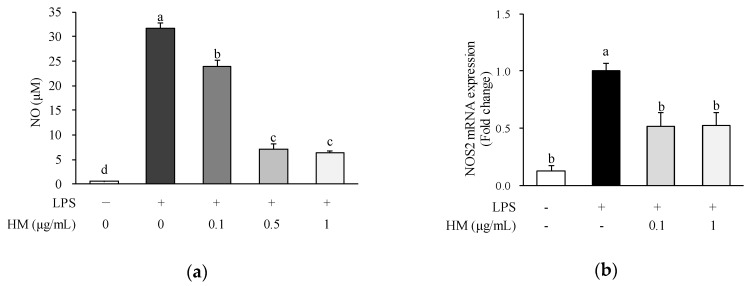
Effects of HM on NO production and NOS2 mRNA expression in RAW264.7 cells. Cells were pre-incubated with indicated concentrations of HM for 1 h and then co-incubated with LPS (1 μg/mL) for 24 h. (**a**) The amount of NO in culture medium was analyzed using a Griess Reagent kit. (**b**) NOS2 mRNA level was determined by qRT-PCR. Values are expressed as mean ± SEM (*n* = 4). Different superscript letters indicate statistical significance (*p* < 0.05). NO, nitric oxide; NOS2, nitric oxide synthase 2; LPS, lipopolysaccharide; HM, high hydrostatic pressure extract of mulberry fruit.

Macrophages play a key role during the early stages of the inflammatory response. LPS, a component of the membrane of gram-negative bacteria, can stimulate the production of inflammatory mediators in macrophages. Once macrophages are activated by various stimuli such as LPS, production of proinflammatory mediators including NO is increased [18]. NO is a signaling molecule that plays a pivotal role in the inflammatory process. While the inflammation progresses, excessive NO is generated by NOS2. Therefore, to investigate the anti-inflammatory effect of HM, we analyzed the NO production and NOS2 expression in LPS-stimulated RAW264.7 macrophages. Since NO in a biological environment is considerably unstable and rapidly oxidizes to nitrite, the nitrite level in the culture medium was determined as an index of NO production. As illustrated in Figure 3a, LPS treatment substantially increased the NO production in the cell supernatant; however, when the cells were treated with HM, at concentrations of 0.1, 0.5, and 1 μg/m, LPS-induced increase of NO production was significantly inhibited in a dose-dependent manner, as shown in Figure 3a. Moreover, the mRNA expression of NOS2 was significantly upregulated in the LPS-treated cells compared to the untreated control, as shown in Figure 3b; however, 0.1 and 1 µg/mL of HM significantly downregulated the mRNA expression of NOS2 compared to the LPS control, as shown in Figure 3b. In a previous study conducted by Qian et al., it has been reported that mulberry fruit dichloromethane extract above 100 μg/mL inhibited both NO production, NOS2 expression, and NF-κB/p65 and pERK/MAPK pathways in macrophages [15]. In addition, oral administration of mulberry water extracts has been reported to cease inflammation by downregulating liver NOS2 expression in a mouse model of liver injury [19,20]. Therefore, it is suggested that HM exerts a protective effect on inflammation by inhibiting NO production and NOS2 mRNA expression in LPS-stimulated macrophages.

### 2.4. Effects of HM on PTGS2 mRNA and Protein Expression

**Figure 4:**
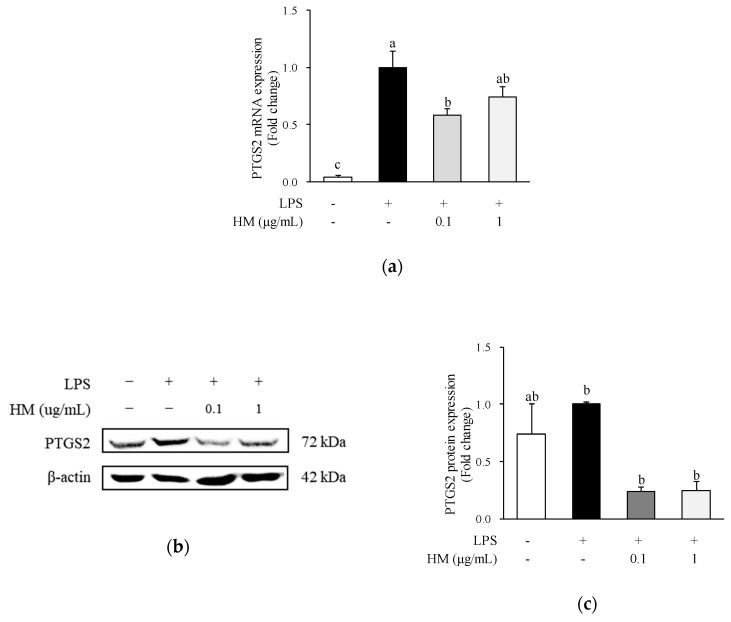
Effects of HM on protein and mRNA expression of PTGS2 in RAW264.7 cells. Cells were pre-incubated with indicated concentrations of HM for 1 h and then co-incubated with LPS (1 μg/mL) for 24 h. (**a**) PTGS2 mRNA levels were determined by qRT-PCR. (**b**) Representative images of western blots. (**c**) PTGS2 protein levels quantified by ImageLab software. Values are expressed as mean ± SEM (*n* = 4). Different superscript letters indicate statistical significance (*p* < 0.05). PTGS2, prostaglandin-endoperoxide synthase 2; LPS, lipopolysaccharide; HM, high hydrostatic pressure extract of mulberry fruit.

Another enzyme that plays a pivotal role in mediating inflammation is PTGS2, also known as cyclooxygenase 2 (COX2). The enzyme is responsible for producing prostaglandin E2 and initiating a variety of proinflammatory processes [21]. Thus, several anti-inflammatory drugs aim to inhibit PTGS2, but their use is limited due to fatal adverse reactions, such as ulceration and perforation of the stomach or intestines [21]. Consequently, various researchers are interested in nontoxic PTGS2 inhibitors. The HM used in this study was derived from natural sources and is therefore readily available and nontoxic. To examine whether HM has an inhibitory effect on PTGS2 expression, we analyzed the mRNA and protein expression of PTGS2. Incubation with LPS significantly increased the mRNA expression of PTGS2 compared to the untreated control cells, as shown in Figure 4a. Nevertheless, 0.1 ug/mL of HM significantly downregulated the mRNA expression of PTGS2 in LPS-stimulated macrophages, as shown in Figure 4a. Moreover, the protein expression of PTGS2 was inhibited by the HM treatment, at concentrations of 0.1 and 1 µg/mL, as shown in Figure 4b,c. A previous study has reported that extract from mulberry (*Morus australis*) leaf decelerates acetaminophen-induced hepatic inflammation, thereby reducing the expression of inflammatory parameters including NOS2 and PTGS2 [22]. Besides the leaves, mulberry fruit dichloromethane extract treatment significantly inhibited LPS-induced upregulation of PTGS2 [15]. Therefore, it is assumed that HM could exert anti-inflammatory effects, presumably due to its ability to suppress PTGS2 mRNA and protein expression in LPS-stimulated RAW264.7 cells.

### 2.5. Effects of HM on Cytokine Production

**Figure 5:**
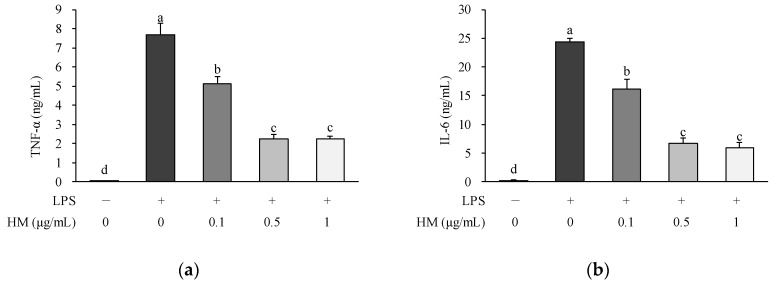
Effects of HM on proinflammatory cytokine production in RAW264.7 cells. Cell were pre-incubated with indicated concentrations of HM for 1 h and then co-incubated with LPS (1 μg/mL) for 24 h. The amounts of (**a**) TNF−α and (**b**) IL-6 in culture medium were determined using ELISA kits. Values are expressed as mean ± SEM (*n* = 4). Different superscript letters indicate statistical significance (*p* < 0.05). TNF−α, tumor necrosis factor−α; IL−6, interleukin 6; LPS, lipopolysaccharide; HM, high hydrostatic pressure extract of mulberry fruit.

Numerous inflammatory cytokines act as the initiators and mediators of the inflammatory response. Among these, TNF-α and IL-6 are the major proinflammatory cytokines released by activated macrophages, and their excessive production has been linked to the development of chronic inflammatory diseases. TNF-α is produced by macrophages in response to bacterial, inflammatory, and other stimuli [23]. TNF-α initiates or contributes to disease pathology by mediating acute inflammatory reactions and chronic inflammation. Additionally, it can stimulate IL-6 synthesis, thereby maintaining the inflammatory response via cytokines with overlapping abilities [24]. Therefore, the selective inhibition of these cytokines may be effectively therapeutic in controlling inflammatory disorders. In our study, the production of proinflammatory cytokines, such as TNF-α and IL-6, was greatly increased in the medium of LPS-treated RAW264.7 cells, compared to the untreated cells, as shown in Figure 5. Nevertheless, the concentrations of TNF-α and IL-6 in the culture medium were dose-dependently reduced in the cells co-treated with HM at concentrations of 0.1, 0.5, and 1 μg/mL compared to the LPS control, as shown in Figure 5. Consistent with our results, in a model of lipopolysaccharide (LPS)-induced sepsis, black mulberry (*Morus nigra* L.) presented anti-inflammatory properties by lowering the serum TNF-α levels [25]. In addition, the combination of mulberry fruit and leaf extracts improved inflammation by suppressing the TNF-α and NOS2 expression in adipose tissue and liver of obese mice [26,27]. Kim et al. have reported that the production of inflammatory cytokines such as IL-6 and TNF-α is accompanied by the expression of NOS2 and PTGS2 induced by inflammatory stimuli, and lowering this inflammatory cytokine production can be effective in inhibiting synergistic induction of NO synthesis in activated macrophages [28]. In previous studies, it has been reported that cyanidin 3-*O*-glucoside, a major anthocyanin found in HM, possesses anti-inflammatory effects [29,30]. Hassimotto et al. reported that the orally administered anthocyanin mixture from wild mulberry and cyanidin-3-glucoside prevent carrageenan-induced peritonitis and paw edema in mice [29]. In addition, it has been revealed that cyanidin-3-glucoside ameliorates the LPS-induced secretion of proinflammatory cytokines including TNF-α and IL-6 in human umbilical vein endothelial cells (HUVECs) [30]. Moreover, flavonols found in HM such as rutin [31], astragalin [32], quercetin [33,34], and kaempferol [35,36] have been reported to have anti-inflammatory effects. Rutin inhibited palmitic acid-induced inflammation in macrophages by suppressing the genes related to endoplasmic reticulum (ER) stress [31], while astragalin inhibited LPS-induced NO production and expression of proinflammatory mediators such as NOS2 and PTGS2 in J774A.1 mouse macrophages [32]. In addition, quercetin effectively inhibited the expression of NOS and PTGS2 and production of TNF-α and IL-6 in RAW264.7 macrophages [33,34]. Moreover, both kaempferol and quercetin dose-dependently inhibited NO production and downregulated NOS2 expression in LPS-treated macrophages [35]. Thus, we suggest that HM suppresses the secretion of inflammatory cytokines including TNF-α and IL-6 in LPS-treated RAW264.7 cells, which may also be an important mechanism in the anti-inflammatory process. Moreover, we assumed that polyphenolic compounds of the HM, including several anthocyanins and flavonols, could contribute to the anti-inflammatory activity.

## 3. Materials and Methods

### 3.1. Cells and Reagents

The RAW264.7 cells were purchased from the American Type Culture Collection (ATCC, Rockville, MD, USA). Dulbecco’s modified Eagle’s medium (DMEM), Dulbecco’s phosphate-buffered saline (DPBS), Penicillin-Streptomycin, sodium pyruvate, and fetal bovine serum (FBS) were obtained from Gibco BRL (Grand Island NY, USA). The Cell counting kit-8 (CCK-8) was obtained from Dojindo Laboratories (Kumamoto, Japan). The Griess Reagent kit was purchased from Invitrogen (Carlsbad, CA, USA). An assay kit for TNF-α and IL-6 was purchased from Biolegend (San Diego, CA, USA). A RiboEx Total RNA solution was obtained (GeneAll Biotechnology (Seoul, Korea). Moloney Murine Leukemia Virus (M-MLV) Reverse Transcriptase kit and AccuPower 2X Greenstar qPCR MasterMix were obtained from Bioneer Co. (Daejeon, Korea). Radioimmunoprecipitation assay (RIPA) buffer, Laemmli’s 5x sample buffer, and PicoEPD Western Reagent were purchased from Elpis Biotech (Daejeon, Korea). The bicinchoninic acid (BCA) protein assay kit was obtained from Thermo Scientific (Pittsburgh, PA, USA). The protease inhibitors cocktail was purchased from Roche (Indianapolis, IN, USA). Mouse anti-PTGS2 monoclonal antibody and peroxidase-conjugated gout anti-mouse IgG were purchased from Santa Cruz Biotechnology (Dallas, TX, USA). Rabbit anti-β-actin polyclonal antibody and peroxidase-conjugated gout anti-rabbit IgG were obtained from Bioss antibodies (Woburn, MA, USA). Pectinex ultra color and Pectinex BE XXL were from Daejong Trade Co. (Seoul, Korea). All other reagents were of analytical grade and were obtained from Sigma–Aldrich (St Louis, MO, USA).

### 3.2. Preparation of HM

The frozen mulberry fruit was purchased from Sang-ju Silkworm Farming Association (Sang-ju, Korea) in February 2017. The HM was prepared by the Korea Food Research Institute (KFRI; Wanju, Korea). Mulberry fruits (500 g) were cut into small particles and homogenized in a Waring blender for 5 min. The mulberry fruit slurry was mixed with 40,000 units each of enzymes Pectinex ultra color and Pectinex BE XXL. The mixtures were then poured into plastic bags, removing excess air, and transferred to a high-pressure apparatus (TFS-50L, Innoway Co., Bucheon, Korea) at 100 MPa for 4 h at 50 °C. The extracts were then boiled for 10 min to inactivate the enzyme. After cooling, the extracts were centrifuged (11,000× *g*, for 5 min) and filtered through Whatman No. 5 filter paper. These extracts were then lyophilized and stored at −20 °C until further use.

### 3.3. UPLC-PDA-Q/TOF-MS Analysis

For analyzing anthocyanins, HM (1 g) was added to 10 mL of 5% formic acid (*v/v*) and was stirred for 24 h. The mixture was centrifuged (3000 rpm, at 4 °C, for 15 min), and then the supernatant was filtered through a polyvinylidene difluoride (PVDF) syringe filter (0.2 μm). The filtrate (0.5 mL) was diluted with 4.5 mL of water. The diluted extract (1 mL) and 100 ppm of cyanidin 3,5-diglucoside (1 mL, internal standard) were loaded onto the Sep-Pak C18 cartridge, washed with 2 mL of water, and eluted from the Sep–Pak cartridge using 3 mL methanol. The extract was concentrated by a stream of nitrogen gas and then resuspended in 0.2 mL of 5% formic acid (*v/v*). For the analysis of flavonols, HM (1 g) was mixed with 10 mL of methanol:water:formic acid (50:45:5, *v/v/v*) solution, containing 20 ppm of galangin (internal standard), and was stirred for 30 min. The mixture was centrifuged (3000 rpm, at 10 °C, for 15 min), and then the supernatant was filtered through a PVDF syringe filter (0.2 μm). The filtrate (0.5 mL) was diluted with 4.5 mL of water. The diluted extract (5 mL) was loaded onto the Sep-Pak C18 cartridge, washed with 2 mL of water, and eluted from the Sep-Pak cartridge using 3 mL of methanol. The extract was concentrated by evaporation using nitrogen gas and then resuspended in 0.2 mL of methanol:water:formic acid (50:45:5, *v/v/v*) solution.

A UPLC-PDA-Q/TOF-MS was used for the analysis of anthocyanins and flavonols presented in HM. The PDA was set at 350 nm (flavonoid) and 515 nm (anthocyanin) and ultraviolet-visible (UV-vis) spectra were recorded from 210 to 600. The analytical equipment and conditions were as follows: column: Kinetex 1.7 μm XB-C18 100 A, 150 × 2.1 mm (Phenomenex, Torrance, CA, USA); precolumn: ACQUTTY UPLC BEH C18, 2.1 x 5 mm, 1.7 μm (Waters Corporation, Milford, MA, USA); mobile phase: solvent A (0.5% formic acid in water) and solvent B (0.5% formic acid in acetonitrile); flow rate 0.3 mL/min; volume of injection 2 μL; column temperature 30 °C; running time 40 min; and gradient condition: 0 min 5% (B), 20 min 25% (B), 25 min 50% (B), 30 min 90% (B), 32 min 90% (B), 35 min 5% (B), and 40 min 5% (B). The mass analysis conditions were as follows: ion source temperature 120 °C (electrospray ionization positive); desolvation temperature 500 °C; desolvation gas flow 1050 L/h; cone gas 50 L/h; capillary voltage 3500 V; sampling cone voltage 40 V; extraction cone voltage 4 V; and mass range 100–1200. Quantification of individual compound levels was calculated using the following formula:

(1) Content (mg/100 g)=((P1÷P2)×C×Dilution factor)/1000×100,

where *P*1 is a peak area of sample, *P*2 is a peak area of internal standard, and C is a concentration of internal standard.

### 3.4. Cell Culture

RAW264.7 cells were cultured in DMEM supplemented with 100 units/mL of Penicillin-Streptomycin, 1 mM of sodium pyruvate, and 10% (*v/v*) heat-inactivated FBS under conditions of 37 °C and 5% CO_2_. The HM was dissolved in DMEM, filtered through a 0.2 um pore size membrane (Sartorius, Gottingen, Germany), and further diluted with DMEM to the tested concentrations. Cells without HM served as a control.

### 3.5. Cell Viability Assay

The cell viability of the HM was assessed with the water-soluble tetrazolium salt (WST)-8 assay using a CCK-8 kit as described previously [37]. RAW264.7 cells were seeded in 96-well plates at a density of 1 × 10^4^ cells/well and incubated for 24 h. After removing the medium, the adherent cells were washed with DPBS and incubated with 0, 0.05, 0.1, 0.5, 1, 5, or 10 μg/mL of HM for 24 h HM in the presence or absence of LPS (1 μg/mL). The optical density at 450 nm was measured using a microplate reader (Varioskan Flash, Thermo Fisher Scientific, Waltham, MA, USA). The results are presented as the percentage of the control.

### 3.6. NO Assay

The concentration of NO was analyzed as nitrite using a Griess Reagent kit according to the manufacturer’s instructions. RAW264.7 cells were plated at a density of 5 × 10^4^ cells/well in 24-well cell culture plates and incubated for 24 h. After removal of the medium, HM at concentrations of 0.1, 0.5, and 1 μg/mL were added to each well and incubated for 1 h. The cells were further co-incubated with 1 μg/mL of LPS for 24 h. Each culture medium (150 μL) was mixed with Griess reagent (20 μL) and distilled water (130 μL) and incubated for 30 min at room temperature (RT; 20–25 °C). The absorbance of the mixture was read at 540 nm using a microplate reader (Varioskan Flash, Thermo Fisher Scientific). The NO production was calculated with a standard curve prepared with NaNO_2_.

### 3.7. Enzyme-Linked Immunosorbent Assay (ELISA) for TNF-α and IL-6

The amount of TNF-α and IL-6 was measured using an ELISA kit according to the manufacturer’s instructions. RAW264.7 cells were plated at a density of 5 × 10^4^ cells/well in 24-well cell culture plates and incubated for 24 h. After removing the medium, HM at concentrations of 0.1, 0.5, and 1 μg/mL were added to each well and incubated for 1 h. The cells were further co-incubated with 1 μg/mL of LPS for 24 h. Post-incubation, the culture medium was collected from each well and stored at −70 °C for the cytokine analysis. Sixty-fold diluted samples were used for detecting TNF-α and IL-6 to not exceed the standard range.

### 3.8. Quantitative Reverse Transcriptase Polymerase Chain Reaction (qRT-PCR)

RAW264.7 cells were seeded at a density of 2.5 × 10^5^ cells/well onto a 6-well culture plate and incubated for 24 h. After removal of the medium, HM at concentrations of 0.1 and 1 μg/mL were added to each well and incubated for 1 h. The cells were further co-incubated with LPS (1 μg/mL) for 24 h. Total RNA was extracted from the cells using RiboEx Total RNA solution. The cDNAs were synthesized from RNA, using a M-MLV Reverse Transcriptase kit. The qRT-PCR was then performed using the AccuPower 2X Greenstar qPCR MasterMix and Rotor-Gene 3000 (Corbett Research, Sydney, Australia). The primer sequences are indicated in Table 3. The delta-delta Ct method was used for relative quantification [38], and β-actin was used as the reference gene for normalization. Values are expressed as fold changes of the control.

### 3.9. Western Blot Analysis

RAW264.7 cells were seeded at a density of 2.5 × 10^5^ cells/well onto a 6-well culture plate and incubated for 24 h. After incubation, HM at concentrations of 0.1 and 1 μg/mL were added to each well and incubated for 1 h. The cells were further co-incubated with 1 μg/mL of LPS for 24 h. For the extraction of total protein from the cell lysates, the adherent cells were detached with a cell scraper with 100 μL of ice-cold RIPA buffer [50 mM Tris-HCl, pH 7.5, 150 mM NaCl, 1% NP-40, 0.5% deoxycholic acid, 0.1% SDS, 1 mM PMSF] containing protease inhibitors. The resulting cell lysates were transferred to a new tube, incubated for 30 min on ice, and then centrifuged (14,000 rpm, at 4 °C, for 20 min). The protein concentration of the extracts was determined using a BCA protein assay kit according to the manufacturer’s instructions. For western blotting, the total protein extracts were mixed with Laemmli’s sample buffer, and equal amounts of denatured proteins (20 μg of protein/lane) were separated using 10% sodium dodecyl sulfate polyacrylamide gel electrophoresis (SDS-PAGE) gels. Proteins were then transferred onto a polyvinylidene difluoride (PVDF) membrane, electrophoretically. After blocking with 5% skim milk in TBST (Tris-buffered saline containing 0.05% Tween-20), the membranes were incubated overnight at 4 °C with antibodies specific for PTGS2 (1:1000) and β-actin (1:1000). The membranes were then incubated with the anti-mouse IgG (1:2000) and anti-rabbit IgG (1:1000) for 1 h at RT. The immunoreactive protein was visualized using a PicoEPD Western Reagent and Chemidoc XRS+ system (Bio-Rad Laboratories, Philadelphia, PA, USA). Band densities were quantified using ImageLab software (BioRad). The densitometric values for protein bands were normalized to values for β-actin, considered as a constitutive internal standard of protein content. The sample value was expressed relatively to the average value for the control group, which was set to 1.0.

### 3.10. Statistical Analysis

Results are expressed as mean ± standard error of the mean (SEM). All experiments were performed in at least three independent experiments. Statistical analyses were performed by SPSS (SPSS, for Windows, version 19; IBM Corporation, Armonk, NY, USA). Significant differences among the groups were determined by one-way analysis of variance test followed by Tukey’s multiple comparison test. A p-value less than 0.05 was considered as statistically significant.

## 4. Conclusions

In conclusion, our results indicate that HM had anti-inflammatory effects on LPS-induced RAW264.7 cells. These effects were partially associated with the inhibition of production of NO and proinflammatory cytokines (TNF-α, IL-6), as well as the expression of NOS2 and PTGS2 in LPS-stimulated RAW264.7 macrophages. It is demonstrated that HM may prevent inflammation by inhibiting the inflammatory mediators and cytokines *in vitro*; however, further studies are necessary to determine whether this is also applicable *in vivo*.

## Figures and Tables

**Table 1:** Contents of total anthocyanins isolated from HM.

Compound	Content (mg/100 g)
Cyanidin 3-*O*-glucoside	75.85 ± 2.79
Cyanidin 3-*O*-rutinoside	49.08 ± 1.92
Pelargonidin 3-*O*-glucoside	2.22 ± 0.06
Total anthocyanins	127.15 ± 4.73

Compounds were detected in positive ion mode ([M + H]^+^) using UPLC-PDA-Q/TOF-MS. Each value was calculated as mean ± standard deviation (SD) of three replicates. HM, high hydrostatic pressure extract of mulberry fruit.

**Table 2:** Contents of total flavonols isolated from HM.

Compound	Content (mg/100 g)
Quercetin 3-*O*-rutinoside (rutin)	8.05 ± 0.19
Quercetin 3-*O*-glucoside (isoquercitrin)	3.14 ± 0.11
Quercetin 3-*O*-(6″-*O*-malonyl)glucoside	1.22 ± 0.03
Kaempferol 3-*O*-rutinoside (nicotiflorin)	0.47 ± 0.03
Kaempferol 3-*O*-glucoside (astragalin)	0.37 ± 0.20
Quercetin	12.99 ± 0.45
Kaempferol	0.54 ± 0.00
Total flavonols	26.79 ± 0.94

Compounds were detected in positive ion mode ([M + H]^+^) using UPLC-PDA-Q/TOF-MS. Each value was calculated as mean ± standard deviation (SD) of three replicates. HM, high hydrostatic pressure extract of mulberry fruit.

**Table 3:** Primers for quantitative reverse transcriptase polymerase chain reaction (qRT-PCR).

Gene ^1^	GenBank Number	Primer Sequences (5′-3′)	Product Size (bp)
β-actin	NM_007393	F: GGACCTGACAGACTACCTCA	208
		R: GTTGCCAATAGTGATGACCT	
NOS2	BC062378.1	F: GCTACTGGGTCAAAGACAAG	191
		R: GCTGAACTTCCAGTCATTGT	
PTGS2	NM_011198.4	F: GAACCTGCAGTTTGCTGTGG	93
		R: ACTCTGTTGTGCTCCCGAAG	

^1^ NOS2, nitric oxide synthase 2; PTGS2, prostaglandin-endoperoxide synthase 2.
